# Diel and spatial variability in cyanobacterial composition, gene abundance, and toxin concentration: a pilot study

**DOI:** 10.1038/s41598-025-18453-5

**Published:** 2025-10-06

**Authors:** V. G. Christensen, L. R. Katona, J. F. LeDuc, R. P. Maki, H. T. Olds, J. C. Smith, H. E. Trompeter

**Affiliations:** 1 U.S. Geological Survey, Upper Midwest Water Science Center, St. Paul, MN USA; 2 Surfrider Foundation, San Clemente, CA USA; 3 Voyageurs National Park, International Falls, MN USA

**Keywords:** Cyanobacteria, Microcystins, Photosynthetically active radiation, eDNA, Anatoxin-a, Saxitoxins, Ecology, Ecology, Environmental sciences, Genetics

## Abstract

We designed a pilot field study to assess relations between sunlight, cyanobacteria, and cyanotoxins. In 2021, we collected day (07:00 h, 10:00 h, 13:00 h, 16:00 h) and night samples (19:00 h, 22:00 h, 01:00 h, 04:00 h) at two locations in Kabetogama Lake, MN, USA. One sample set was collected from the lakeward end of a boat dock and the other on the nearby shoreline. Cyanobacterial phylogenetic eDNA differences over 24 h (pseudo F = 2.0938, *p* = 0.127) were not significant. Copies of anatoxin (*anaC*) and microcystin (*mcyE*) synthetase genes varied significantly over the sampling times at the dock (Friedman Χ^2^ = 15.01, *df* = 7, *p* = 0.036; Friedman Χ^2^ = 19.22, *df* = 7, *p* = 0.008) and the shoreline (Friedman Χ^2^ = 19.33, *df* = 7, *p* = 0.007; Friedman Χ^2^ = 20.56, *df* = 7, *p* = 0.005), with the highest *anaC* counts occurring during the night for both sites. Additionally, the highest total and dissolved microcystin concentrations occurred at night. Despite the proximity of the sampling locations, cyanobacterial phylogenetic eDNA results indicate that the variability between sites (pseudo-F = 27.547, *p* = 0.001) were greater than temporal differences over 24 h (pseudo F = 2.0938, *p* = 0.127). Understanding the effect of diel and spatial variability may help researchers and resource managers make informed decisions about sampling and potential exposure.

## Introduction

In aquatic environments, rapid reproduction and accumulation of cyanobacteria can lead to the formation of cyanobacterial blooms^1–3^. These blooms can restrict recreation^4^deplete dissolved oxygen^5,6^ and result in fish mortality^7^. More importantly, some cyanobacteria can produce toxic metabolites, called cyanotoxins^3^ that may adversely affect humans, animals, and ecosystems. Emerging technologies allow for rapid detection of cyanobacterial accumulation (remote sensing)^8^ or cyanotoxin presence (test strips)^9^ but water resource managers may rely on discrete water sample collection and analysis using a variety of methods to understand the condition of water bodies. Cyanobacterial blooms are often patchy and can form or dissipate rapidly^10^ which can complicate sample collection and potentially underrepresent risks if samples do not represent overall water body conditions.

### Temporal variation

The main purpose of this pilot study was to examine temporal variation in cyanobacterial composition, gene abundance, and toxin concentration. Cameron et al.^11^ studied shallow, well-mixed lakes and showed that cyanobacteria varied in samples collected in the morning, midday, and afternoon.

The stability of the water column is a factor in temporal variation, which can challenge toxin detection if the system dynamics, such as oscillatory diel variation, are not considered in the sampling design^11^. Many cyanobacteria can control their buoyancy and move through the water column freely. *Microcystis* may maintain position in the photic zone, moving where nutrients, light, and stratification are favorable. Vertical migration of *Microcystis*, which requires buoyancy^12^ allows nutrient uptake from lower in the water column and light from higher in the water column. Movement is likely distinctive to individual taxa, depending on differences in cell structure and size^13^. Additionally, buoyancy of individual cyanobacteria can change throughout 24 h, as actively photosynthesizing cells produce carbohydrates during the day, become denser, and start to sink^14^. During darkness, carbohydrates are consumed, CO_2_ is produced, and cells begin to rise as a result of increased buoyancy^14,15^. Thus, the effect of daily light and dark cycles on photosynthesis are an important part of the temporal variation in cyanobacteria.

Research suggests there may be a time-of-day element to the risk of toxin exposure that goes beyond the movement of cyanobacteria. Diurnal fluctuations in alkaloid levels have been observed for stationary plants such as hemlock (*Conium maculatum*), deadly nightshade (*Atropa bella-donna*), and opium poppies (*Papaver somniferum*); these fluctuations may be responsible for the rules of herb gathering and drug harvesting in ancient times^16^. For cyanotoxins, laboratory tests have demonstrated that anatoxin-a (an alkaloid) undergoes rapid degradation to non-toxic forms in sunlight^17–19^ and at elevated pH^17^ which are common conditions in the late summer months when blooms and toxins occur in northern temperate climates^20^. However, these laboratory studies were performed on dissolved anatoxin-a highlighting a research gap concerning intracellular anatoxin-a.

Intracellular anatoxin and saxitoxin has been measured at high concentrations presumably during daylight hours^21,22^ and the half-life of extracellular anatoxin-a is about 1–2 h under expected light conditions of a decaying bloom in most northern temperate climates^19^. However, the only suspected human death from anatoxin-a in the United States occurred following a swim after dusk^23^. One researcher showed that ultraviolet-B (UVB) radiation (at pH 7) reduced anatoxin-a by 82% in 1 h. When exposed to visible light only, or photosynthetically active radiation (PAR), anatoxin-a showed slight degradation^17^. This degradation of anatoxin-a indicates that concentrations may be underestimated when samples are only collected in daylight. Other cyanobacterial neurotoxins, like saxitoxin, are understudied in freshwater^24^ and we found little information on how this neurotoxic alkaloid may be affected by sunlight or PAR. One study showed degradation of dissolved, extracellular saxitoxin when exposed to natural and stimulated sunlight, indicating that toxin concentrations may be greater during darkness hours^25^.

### Spatial variation

While examining the temporal, or more specifically diel, variability was the main goal of this research, spatial variation also was considered. The distribution of taxa is dependent on hydrology and morphology, and meteorological variables such as wind can have a substantial effect on the location of cyanobacteria within a lake^26^. Higher near-shore concentrations are typically caused by wind-driven accumulations of cyanobacteria^10,26^. A study by Wu et al.^27^ found that the accumulation of *Microcystis* was primarily determined by surface drift, which requires the cyanobacteria to be high enough in the water column to be subject to the winds. Many field studies collect samples from the end of a dock or shoreline, and a comparison of spatial results between these two types of sites may be valuable for choosing suitable sites for other studies.

### Cyanotoxins in Voyageurs National Park

Our study took place in Voyageurs National Park (USA), along the USA-Canada border in northern Minnesota. Voyageurs is a water-based park, and most park visitors engage in water-based activities, such as boating, swimming, fishing, and water sports. Visitors need a boat to reach all park campsites and they spend over 700,000 h per year angling on the largest bodies of water in the park^28^ sometimes at night or during pre-dawn hours.

Previous research demonstrated that the cyanotoxins anatoxin-a, microcystin, and saxitoxin have been present in the recurring blooms at recreational areas within Kabetogama Lake, one of Voyageurs National Park’s most popular water bodies^29,30^. However, until the current study, samples at the park have only been collected midday^31^.

Microcystin concentrations have been detected at levels of concern during the day^32^ but neurotoxins have not (a suggested recreational health level is ~ 60 µg/L for anatoxin-a and ~ 30 µg/L for saxitoxin)^3^. Dissolved-phase anatoxin-a concentrations may be lowest late in the day due to photodegradation under sunlight^19^ particularly when cell densities do not induce self-shading to protect the toxin. Additionally, diel cycles in cyanobacteria, involving light-dependent changes in cell physiology and metabolism, may influence toxin production and release^33,34^. If neurotoxins are present at levels of concern during the night, resource managers may be unaware of health risks posed to visitors engaging in popular activities such as camping, fishing, or staying overnight in houseboats in this water-based park.

Studies have shown that microcystin concentrations tend to decrease in samples collected during the night relative to samples collected during daylight^35^. Transcriptomic data analyzed by Wang and Mou^36^ support this finding and show a decrease in microcystin biosynthesis genes during dark relative to light conditions. Despite the field evidence that microcystin has a diel component and laboratory and statistical evidence that indicate a time-of-day relation to neurotoxins, few field studies have evaluated the diel variation in the neurotoxin anatoxin-a^37^ and we know of no field studies that have evaluated saxitoxin concentrations throughout the hours of darkness in a natural system.

**Fig. 1:**
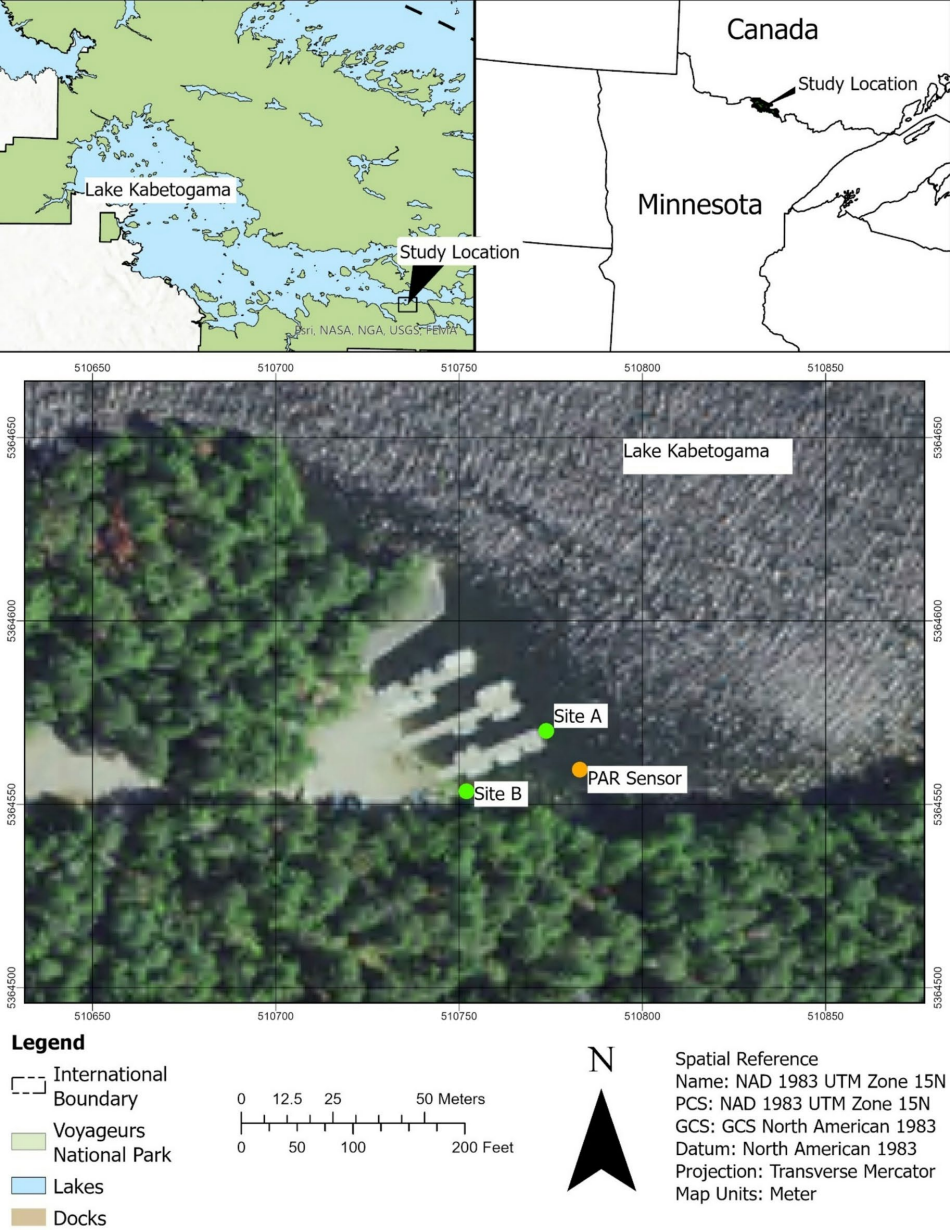
Map of study site, including collection locations for dock samples (site A), adjacent shoreline samples (site B), and photosynthetically active radiation (PAR) measurements near the Ash River Boat Docks on Kabetogama Lake, MN (USA) on Sept. 9–10, 2021. The two sites are less than 30 m apart. Figure was produced in ArcGIS Pro (v. 3.3.1) using satellite imagery from https://naip-usdaonline.hub.arcgis.com/^39^.

In preparation for two related comprehensive studies (temporal and spatial) of cyanotoxins at recurring bloom sites in Kabetogama Lake, Voyageurs National Park, we began a pilot study whereby we collected samples over 24 h at two locations (Fig. 1) during a visually continuous bloom in September 2021. Daylight samples were collected at about 07:00 h, 10:00 h, 13:00 h, 16:00 h and night samples at about 19:00 h, 22:00 h, 01:00 h, 04:00 h. The collection date represented a time of year when day hours and night hours are roughly equal (12 h each) and when blooms are frequently present on the study lake. Site selection was critical because the composition and toxicity of a single bloom varies, particularly during active blooms^38^. We hypothesized that cyanotoxin gene abundance, cyanobacteria composition, and toxin concentration would be highly variable across 24 h.

## Results

### Temporal variation

A PERMANOVA analysis using Bray-Curtis index showed that the differences between samples collected during the day were not significantly different than samples collected during the night in terms of the entire phytoplankton communities (pseudo F = 1.2473, *p* = 0.308) or when considering only the cyanobacteria portion of the communities (pseudo F = 2.0938, *p* = 0.127).

**Fig. 2:**
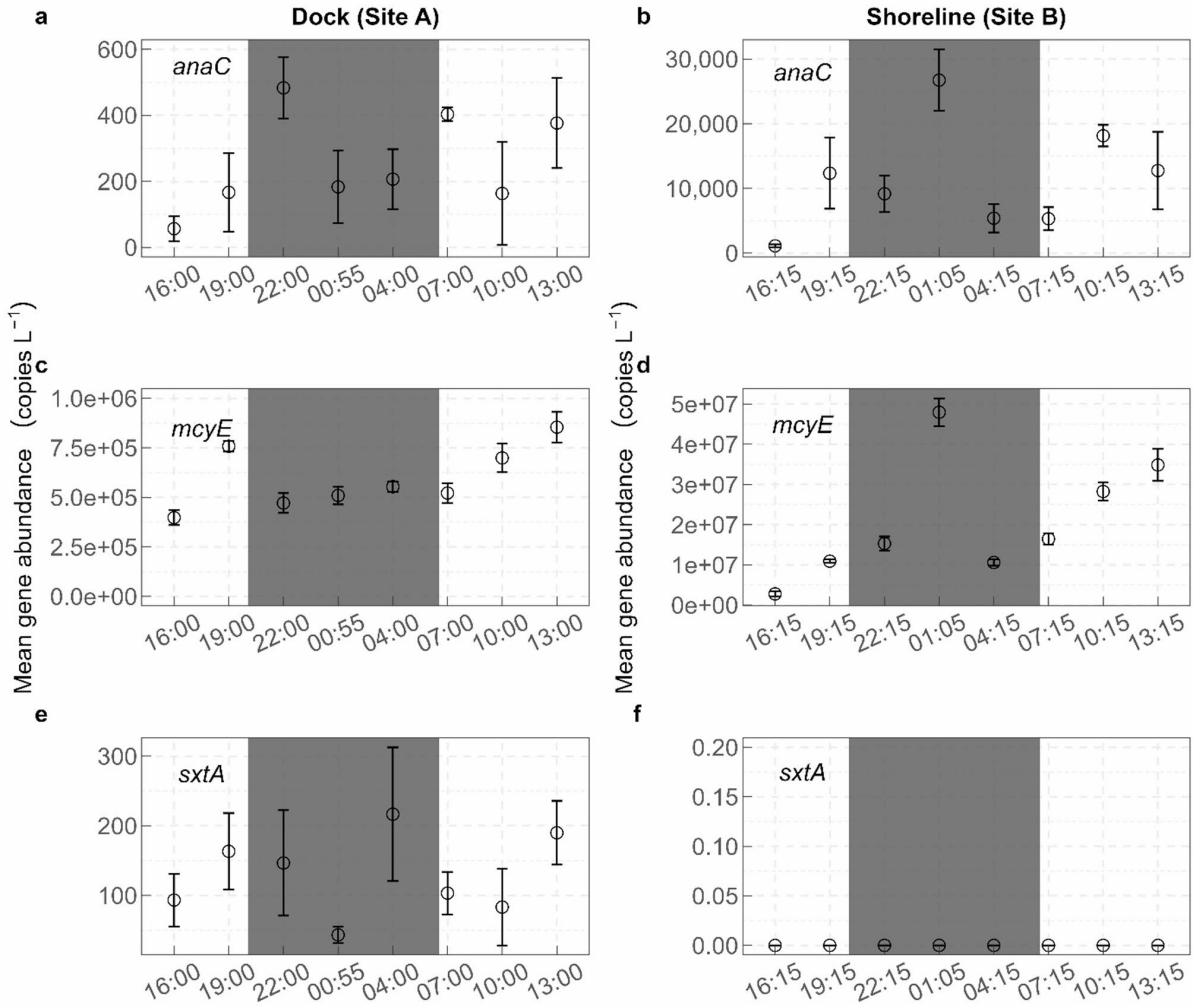
Mean abundance of synthetase genes in water samples over 24 h collected at Ash River Boat Docks on Kabetogama Lake, MN, September 9–10, 2021: (**a**) anatoxin (*anaC*) at the dock (site A), (**b**) *anaC* at the shoreline (site B), (**c**) microcystin (*mcyE*) at the dock, (d) *mcyE* at the shoreline, (e) saxitoxin (*sxtA*) at the dock, and (f) *sxtA* at the shoreline. Points represent the mean cyanotoxin gene abundance and error bars are one standard deviation about the mean. Note the difference in scale for each cyanotoxin gene between sites.

Copies of the anatoxin synthetase gene (*anaC*), the microcystin synthetase gene (*mcyE*), and the saxitoxin synthetase genes (*sxtA*), showed some temporal variation (Fig. 2), but other than *mcyE*, which gradually increased throughout the sampling event at the dock site (Fig. 2c) and at the shoreline with one outlier (Fig. 2d), there are no consistent increasing or decreasing trends in *anaC* or *sxtA* through time. However, it is notable that the highest *anaC* counts occurred during the night for both sites (Fig. 2a and b), although the error bars for one site (site A, Fig. 2a) overlap with two daytime samples. Microcystin synthetase genes were the most abundant cyanotoxin synthetase genes across both sites (mean ± standard deviation across all samples = 59,570 ± 15,623 and 2,088,904 ± 1,440,424 copies/L at the dock and shoreline site, respectively). While there is high variability in qPCR measurements and there should be caution against using these qualitatively^40^the values are useful for comparison to the other toxin genes. For example, the abundances of *anaC* were relatively low at the dock site (229 ± 161 copies/L) when compared with the shoreline site. Abundances of *sxtA* at both sites (127 ± 75 and 0 copies/L at the dock and shoreline site, respectively) were negligible. Abundance of *anaC* varied significantly over the sampling times at the dock (site A; Friedman Χ^2^ = 15.01, *df* = 7, *p* = 0.036) and the shoreline (site B; Friedman Χ^2^ = 19.33, *df* = 7, *p* = 0.007). Significant differences in *anaC* abundance occurred at the shoreline between the 01:05 h sampling time and the 16:15 h (*p* = 0.008), 04:15 h (*p* = 0.033), and 07:15 h (*p* = 0.044) sampling times. Abundance of *mcyE* gene varied significantly over the sampling times at the dock (site A; Friedman Χ^2^ = 19.22, *df* = 7, *p* = 0.008) and shoreline (site B; Friedman Χ^2^ = 20.56, *df* = 7, *p* = 0.005). Significant variation in *mcyE* abundance occurred between the 16:00 h and 19:00 h (*p =* 0.04), the 16:00 h and 13:00 h (*p* = 0.01), and the 22:00 h and 13:00 h (*p =* 0.03) sampling times at the dock (site A). At the shoreline (site B), *mcyE* varied significantly between 01:05 h and the 16:15 h (*p* = 0.008), 19:15 h (*p* = 0.03), and 04:15 h (*p =* 0.04) sampling times. There was also significant variation in *mcyE* abundance between the 16:15 h and 13:15 h sampling time at site B (*p =* 0.03). We did not detect significant variation in *sxtA* abundance among sampling times at the dock (site A; Friedman Χ^2^ = 13.47, *df* = 7, *p* = 0.06) and *sxtA* was not detected in any samples from the shoreline (site B).

**Fig. 3:**
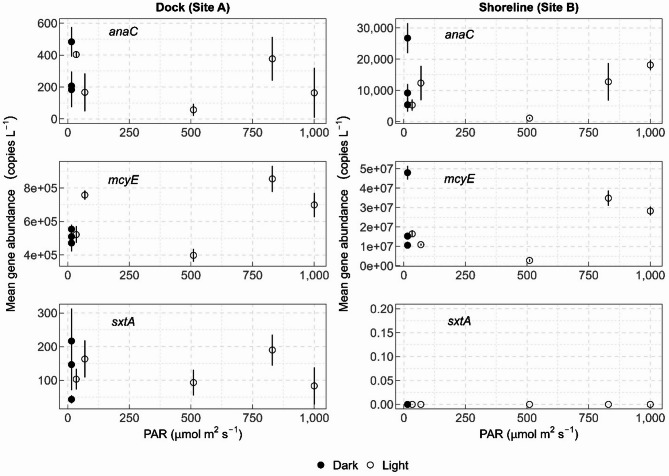
Mean gene abundance of anatoxin synthetase genes (*anaC*), microcystin synthetase genes (*mcyE*), and saxitoxin synthetase genes (*sxtA*) in water samples compared to photosynthetically active radiation (PAR) data collected from the end of a dock (site A) and the adjacent shoreline (site B) over 24 h, at Ash River Boat Docks on Kabetogama Lake, MN, September 9–10, 2021. Note the differences in scale between sites for gene abundances.

When compared to photosynthetically active radiation (PAR), gene abundance appeared to decrease in the mid-PAR range of 510 micromoles per square meter per second (µmol/m^2^/s; Fig. 3), which occurred in the samples collected near 16:00 h. The highest gene abundances occurred below 50 and above 800 µmol/m^2^/s.

**Fig. 4:**
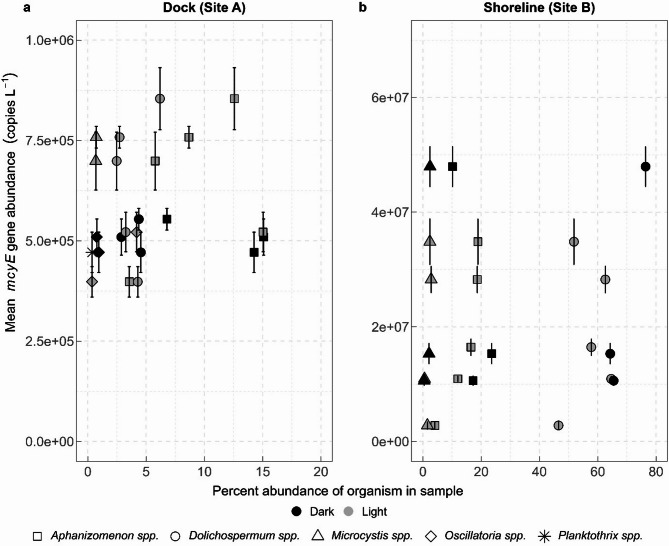
Mean microcystin synthetase (*mcyE*) gene abundance in water samples collected from (**a**) the end of a dock (site A) and (**b**) the adjacent shoreline (site B) at Ash River Boat Docks on Kabetogama Lake, MN, September 9–10, 2021, compared to percent abundance of selected cyanobacterial genera in samples. Note the differences in scales between sites for gene and organism abundance.

A comparison of *mcyE* gene abundance to percent abundance of organisms in a sample revealed that there was higher *mcyE* overall, and particularly during the day at the dock (site A; Fig. 4a), which corresponded with a bloom dominated by *Aphanizomenon* spp. The shoreline site was dominated by *Dolichospermum* spp. in samples collected both during the day and at night, although the highest *mcyE* abundance at the shoreline (site B) corresponded with all three primary cyanobacteria (*Aphanizomenon*, *Dolichospermum*, and *Microcystis*, Fig. 4b).

**Fig. 5:**
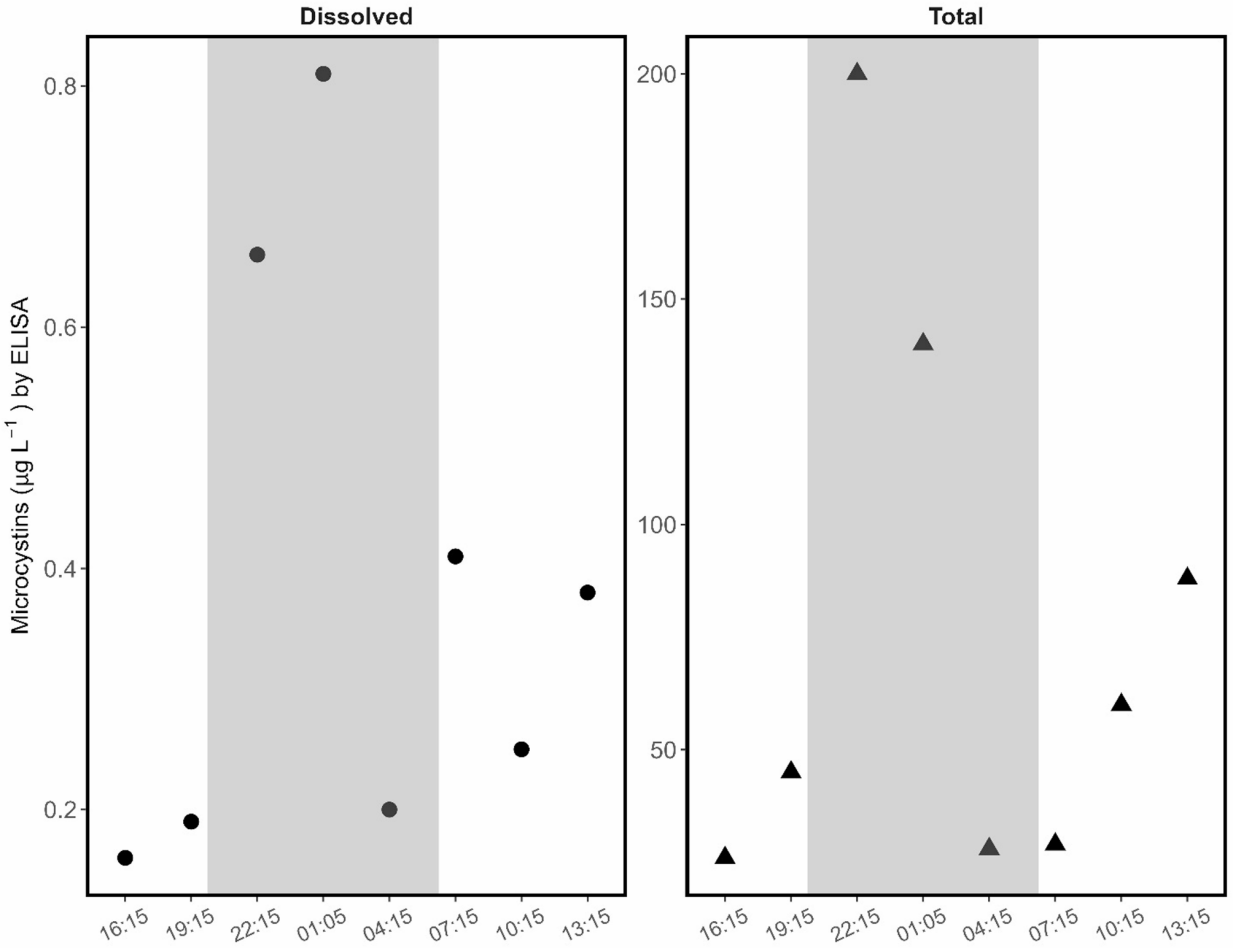
Dissolved (circles) and total (triangles) microcystin by enzyme-linked immunosorbent assay (ELISA) results in water samples collected from the shoreline site (site B) at Ash River Boat Docks on Kabetogama Lake, MN, September 9–10, 2021. Note the differences in scales between dissolved and total concentrations.

Dissolved and total microcystin concentrations are available for the shoreline (site B) only (Fig. 5). The highest concentrations of both dissolved and total microcystin occurred at night during the 22:00 h (0.65 and 200 µg/L, respectively) and 01:00 h (0.70 and 140 µg/L, respectively) sampling times. Conversely, the 04:00 h sample had some of the lowest microcystin concentrations (0.18 and 28 µg/L, respectively) despite also being collected in darkness. This aligned somewhat with the microcystin gene abundances (high levels at 22:00 h and 01:00 h, lower at 04:00 h).

### Spatial variation

**Fig. 6:**
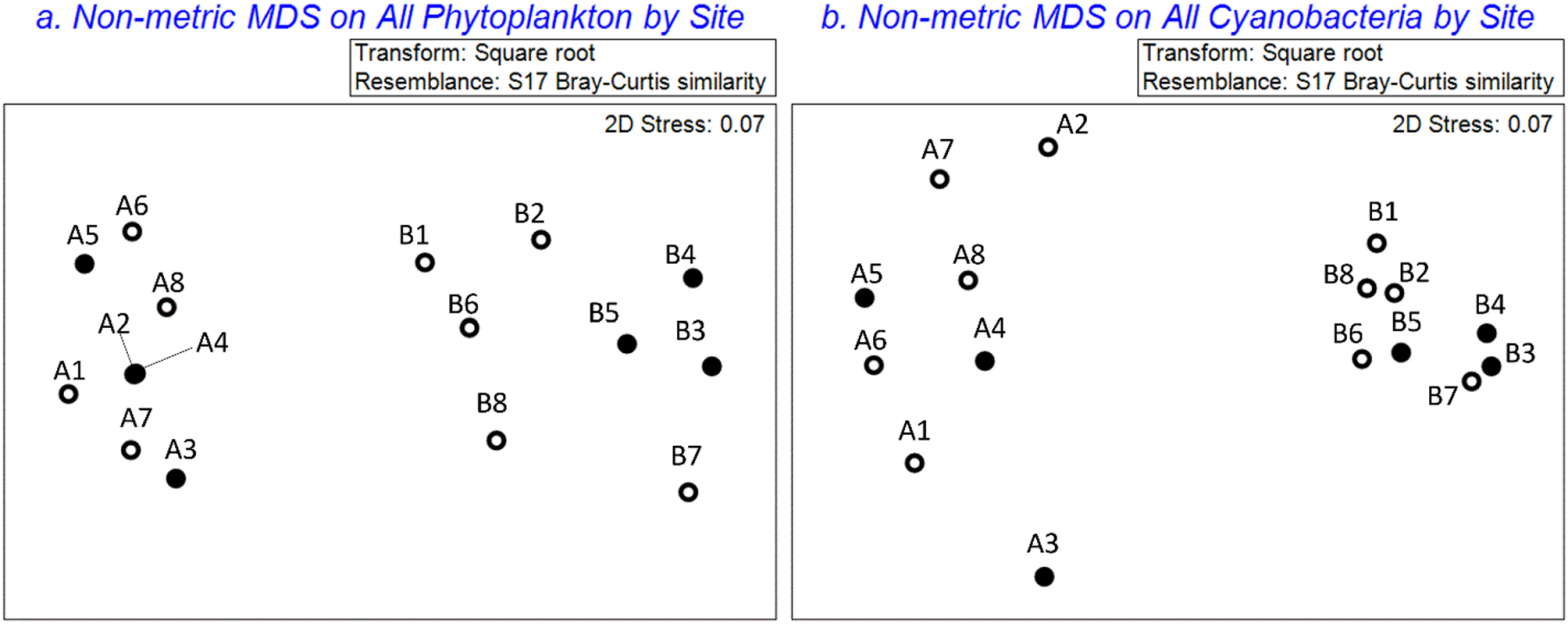
Non-metric multidimensional scaling plots of (**a**) all phytoplankton and (**b**) cyanobacteria only, show less similarity between samples collected at the dock (site A) and adjacent shoreline (site B) than between day and night (open and shaded circles, respectively). Samples were collected from Ash River Boat Docks on Kabetogama Lake, MN, September 9–10, 2021.

While a comparison between the two different sites and collection methods was not the primary focus of this article, the comparison reveals some interesting patterns. Despite their proximity, the phytoplankton collected at the end of the dock (site A) was distinctly different than water collected near the adjacent shoreline (site B) based on Bray Curtis indices (as defined by Clarke and Warwick^41^) on phylogenetic eDNA data (Fig. 6A). This distinct spatial difference was greater than temporal differences. When only cyanobacteria are considered (Fig. 6B), the pattern changes slightly, with greater similarity in the cyanobacteria samples from the shoreline (site B) than at the dock (site A). A permutation multivariate analysis of variance (PERMANOVA) on this data revealed significant differences in the phytoplankton community (pseudo-F = 16.431, *p* = 0.001) and the cyanobacteria community (pseudo-F = 27.547, *p* = 0.001) between the two sites.

**Fig. 7:**
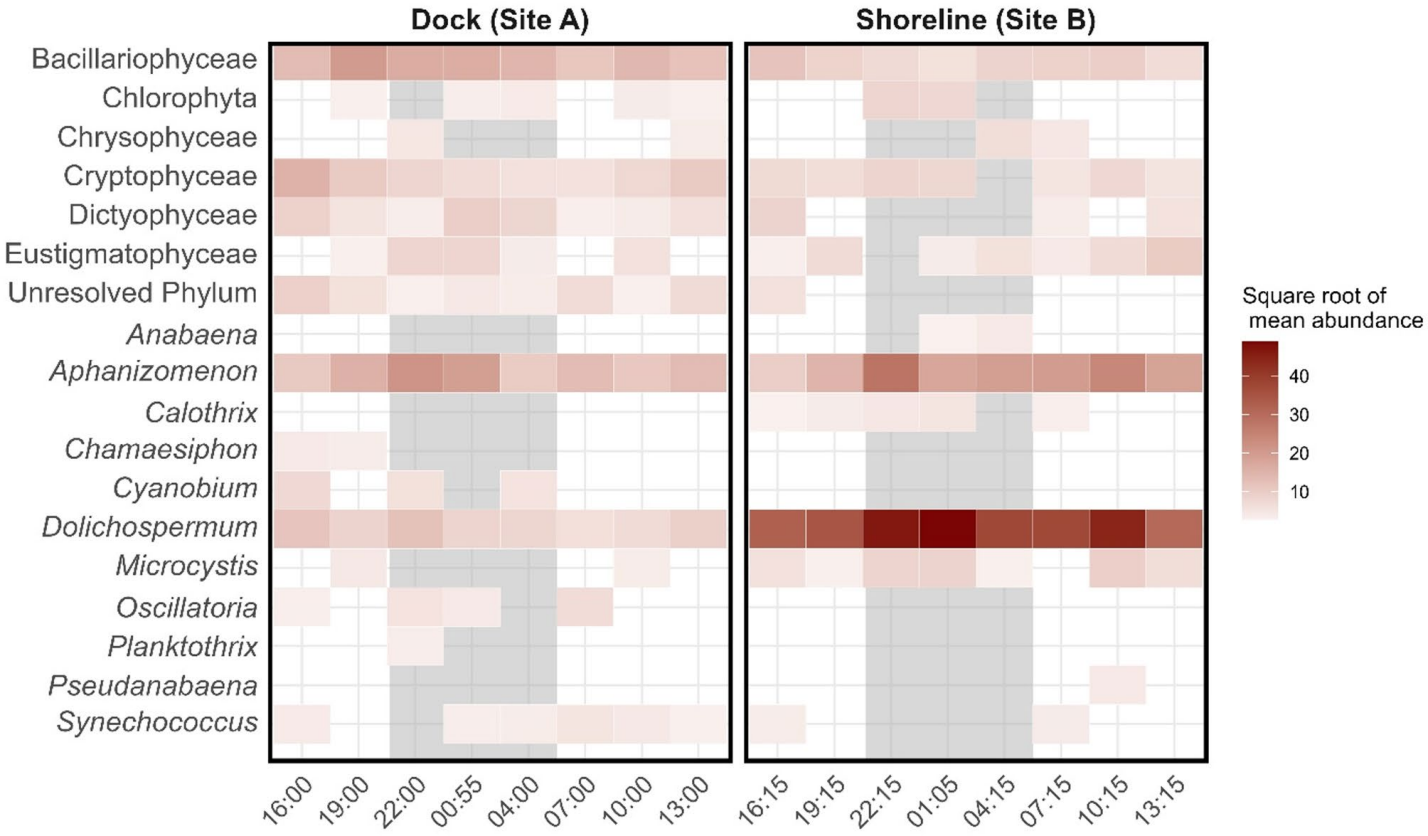
Shade plot of phytoplankton classes in samples collected from Ash River Boat Docks on Kabetogama Lake, MN at the end of a dock (site A) and near the adjacent shoreline (site B), September 9–10, 2021. Gray shading corresponds to night samples (collected during darkness).

The dock (site A) generally had a more diverse range of phytoplankton than the shoreline (site B), with averages of 20 taxa versus 14 taxa. The dock samples also had more Bacillariophyta (diatoms) and fewer putative toxin producers (for example, *Microcystis* and *Dolichospermum*, Fig. 7).

**Fig. 8:**
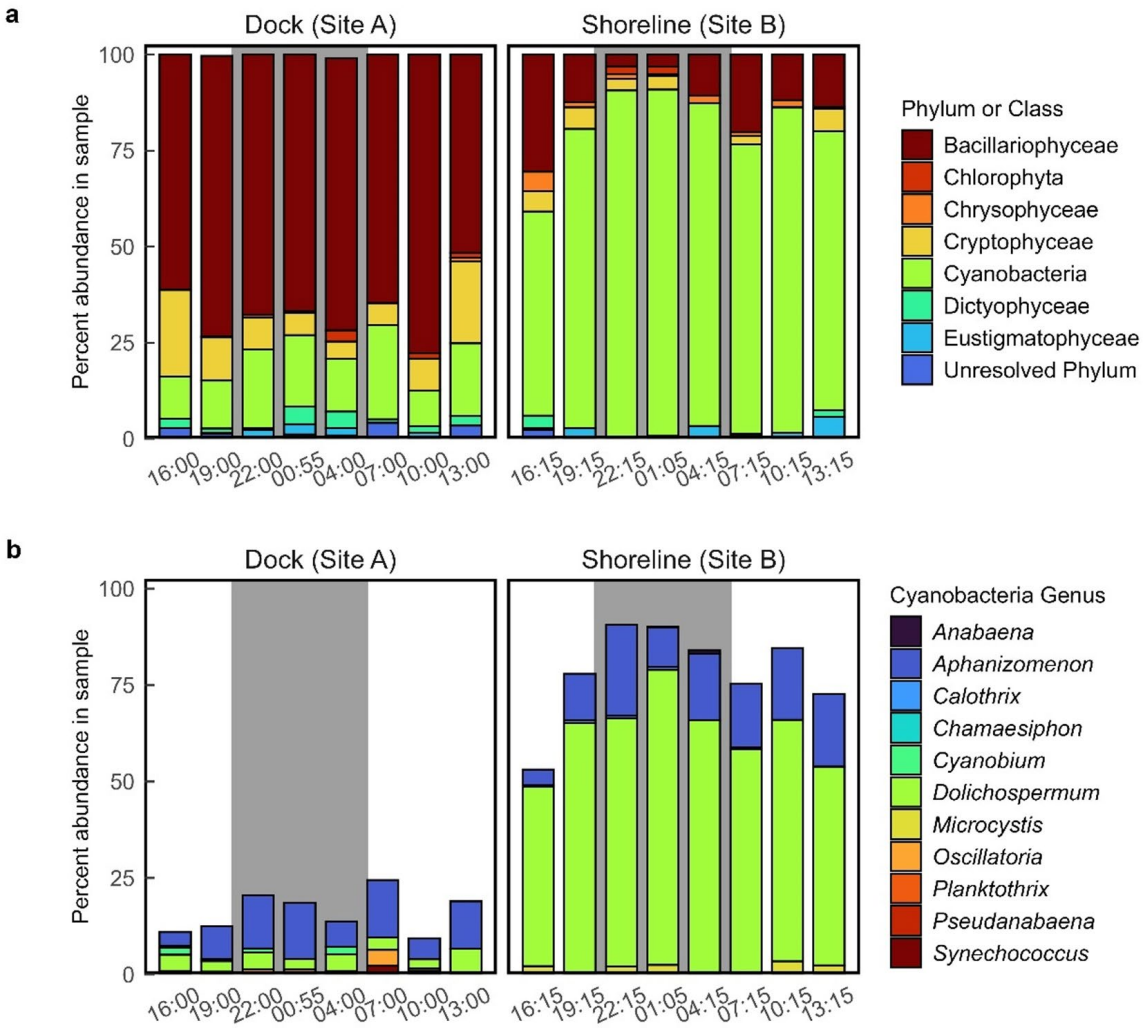
Percent abundance of (**a**) phytoplankton phyla or classes in samples collected from Ash River Boat Docks on Kabetogama Lake, MN at the end of a dock (site A) and near the adjacent shoreline (site B), September 9–10, 2021, and (**b**) Percent abundance of cyanobacteria genera in samples collected from the end of a dock (site A) and near the adjacent shoreline (site B) at Ash River Boat Docks on Kabetogama Lake, MN, September 9–10, 2021. The color scheme in the legend corresponds to the color order of the stacked bars.

Additionally, eDNA analysis revealed substantial differences in percent abundance of the different phyla by site (Fig. 8a). The dock (site A) had substantially more Bacillariophyta (diatoms), whereas the shoreline (site B) had an abundance of cyanobacteria. Abundances varied over the 24-hour period, but this variance was not as great as the differences between sites. Small amounts of Chlorophyta (green algae) were present in 5 of 8 dock (site A) samples throughout the 24 h period, whereas Chlorophyta were only present in the shoreline samples that were collected at night.

In terms of cyanobacteria, percent abundance was substantially different between the dock (site A) and shoreline (site B; Fig. 8b). The most abundant cyanobacterial taxon at the dock site (site A) was *Aphanizomenon* spp., while the most abundant cyanobacterial taxon at the shoreline (site B) was *Dolichospermum* spp. Certain species of *Aphanizomenon* are known to produce both anatoxin-a and saxitoxin^24^. *Dolichospermum* is better known as a microcystin producer^42^.

The cyanotoxin synthetase genes, *anaC* and *mcyE*, were more abundant at the shoreline (site B; Fig. 2b and d) than at the dock (site A, Fig. 2a and c). Conversely, *sxtA* was present in all samples from the dock (site A; Fig. 2e) but was not detected at the shoreline (site B; Fig. 2f).

For toxin results, a spatial comparison could not be made because these data are only available for the shoreline (site B).

## Discussion

Predicting when the risk of cyanotoxin exposure will be greatest is difficult due to lack of information on the variability of toxin production and degradation in a natural system. This pilot project supports a larger temporal study to determine whether potent neurotoxins are a risk during daylight or night hours and a larger spatial study to determine the variability of cyanobacterial composition, gene abundance, and toxin concentration across a bloom.

### Temporal variation

Generally, we found the highest detections of cyanotoxin synthetase genes and dissolved and total microcystin concentrations occurred at night. Phylogenetic eDNA data indicate some differences between the day and night samples, but these were clearly not as substantial as the differences between the sites (Fig. 6). While this pilot study only considered samples collected over 24 h, there is likely a seasonal component as well. Christensen et al.^20^ showed greater similarity among Kabetogama Lake phytoplankton community samples collected during the same season (at three sites > 2 km apart) than between sites.

Gene copies of *anaC*, *mcyE*, and *sxtA*, were variable across the 24 h (Fig. 2), with *mcyE* gene copies at the dock (site A) gradually increasing throughout the sampling event (Fig. 2c). While this evidence supports a diel component in toxin production, other environmental or biological factors could affect the number of *anaC*, *mcyE*, and *sxtA* gene copies.

The highest *anaC* counts occurred during the night for both sites (Fig. 2a and b), although the variation at the dock site (Fig. 2a) make conclusions difficult. The highest *sxtA* count also occurred at night (Fig. 2E; there was no *sxtA* detected at site B throughout the 24 h). Gene abundances documented previously at Kabetogama Lake did not show a significant relation to PAR, but the samples were all collected mid-day^30^. Surprisingly, gene abundance was lower in the mid-PAR range of 510 micromoles per square meter per second (µmol/m2/s; Fig. 3), which were the samples collected near 16:00 h. It could be that there was cloud cover present around this time, indicating the importance of considering PAR data as well as time-of-day in diel studies. Additionally, the diel cycle of photosynthesis may be partially responsible for the relationship between PAR and gene abundance. When photosynthesizing cells produce carbohydrates during daylight, they become denser, and start to sink^14^. Because the mid-PAR ranges in our study occurred in the afternoon (16:00 h), and this was the time of lowest gene abundance, it may be that many of the cyanobacteria capable of producing toxins had moved to other parts of the water column.

The toxin data available for this study is not a complete picture but does include dissolved and total microcystin concentrations at the shoreline site. Interestingly, the highest concentrations of both total and dissolved microcystin occurred at the 22:00 h and 01:00 h sampling times, which is similar to the diel pattern seen with the microcystin gene abundances (high levels at 22:00 h and 01:00 h, lower at 04:00 h). This is inconsistent with other studies, which showed higher microcystin concentrations in the afternoon^15,43,44^ than at other times.

One study that found high light intensity (PAR) was related to microcystin^45^ however, for our pilot study the highest PAR (550–1,000 µmol/m^2^/s) values corresponded to only moderate total microcystin values (26–92 µg/L). The highest total microcystin values (140 and 200 µg/L) corresponded to the lowest PAR values (15 µmol/m^2^/s for both samples). Microcystin degradation can depend on light penetration^46^ so future studies could measure sub-surface PAR when samples are collected at depth.

Although anatoxin-a data is not available for this pilot study, at least one study showed that the rate of photolysis of anatoxin-a depends on sunlight intensity^19^—with anatoxin-a decaying more rapidly the higher the intensity. While it is likely that anatoxin-a and microcystin behave differently when exposed to sunlight, more diel studies are needed to confirm our findings.

This study could be repeated over several days to rule out weather or other variables that may have an effect on community composition, toxin gene abundance, or toxin concentrations.

### Spatial variation

During our pilot study, we found substantial differences in organism abundances between sampling locations despite their proximity (< 30 m apart) and lack of significant wind or other disturbance during sampling. Composition of the phytoplankton communities were different, with a more diverse assemblage of taxa at the end of the dock than at the shoreline site. The dock samples also had fewer putative toxin producers and more Bacillariophyta (diatoms), which in freshwater are generally considered non-toxic and a more nutritious food source for zooplankton than cyanobacteria^47^. Diatoms may have been more abundant at the dock site because stratification may play a role in diatoms establishing deeper in the water column^48^ or nearshore areas may be more turbid and not conducive to diatoms. Diatoms may prevent the formation of cyanobacterial harmful algal blooms (and perhaps some nitrogen-dependent cyanobacteria), because diatoms store nitrogen^48^. Chlorophyta (green algae) were present in 5 of 8 samples at the end of the dock (site A), which was distinctly different than at the shoreline (site B), where Chlorophyta was only present in samples collected at night (after dark; 22:00 h and 01:00 h). Similar to diatoms, Chlorophyta at the dock site may be affected by stratification; lack of Chlorophyta at the shoreline may be due to turbidity.

This difference in composition may be partially attributed to the collection methods and the buoyancy of the phytoplankton in question. The samples from the end of the dock were collected 1 m below the surface with a Van Dorn sampler, and thus may have contained taxa that can moderate their buoyancy, such as *Aphanizomenon*, which can move deeper in the water column to obtain needed nutrients^49^. Chlorophytes also have the ability to regulate their buoyancy^50^ Samples collected below the surface or depth integrated samples may under-represent blooms that form surface scums^51^. Although there was very little wind during this pilot study, the samples at the end of the dock may have been exposed to slightly more wind and mixing; increased mixing can cause a switch from cyanobacteria to diatom dominance^52^. Cameron et al.^11^ discussed the stability of the water column as a factor in diel variability and the role of wind. It is possible that wind played a role in the variability at our shoreline site in Kabetogama Lake because an earlier cyanotoxin mixture model identified wind direction and speed as a significant factor in determining toxin concentrations^30^. However, wind speed during sampling was very low in this protected bay and the effect of wind at either site was likely minimal.

The shoreline samples were collected using grab sampling procedures^26^ which gather surface accumulations. Low turbulence, which was the condition of the shoreline during sampling, may enable buoyant cyanobacteria to float to the surface^53^ and be subject to wind-blown accumulation. The dominant taxon at the shoreline site was *Dolichospermum* spp., a small planktonic taxon that can join together in longer filaments. *Dolichospermum* blooms are often associated with surface scums and reduced diversity of phytoplankton^53 ^perhaps because they block out the light, outcompeting other cyanobacteria.

In terms of cyanotoxin synthetase genes, the most notable difference between sites was the presence of *sxtA* in all samples from the dock (site A) and a complete absence of *sxtA* at the shoreline (site B). Common *sxtA* producers include certain strains of *Aphanizomenon*^24^ a taxon that was dominant at the dock (site A). Some strains of *Dolichospermum* have also been shown to produce saxitoxin^53,54^ such as *D. circinale*^55^ and *D. lemmermannii*^56^ and while *Dolichospermum* was dominant on the shoreline, these strains were not identified in the data set. The *anaC* and *mcyE* genes were higher at the shoreline (site B) than at the dock (site A). Anatoxin producers include both *Aphanizomenon* and *Dolichospermum*^24^ and numerous taxa have the capability of producing microcystin, including *Anabaena*, *Aphanizomenon*, *Calothrix*, *Dolichospermum*, *Microcystis*, *Oscillatoria*, *Pseudanabaena*, *Planktothrix*, and *Synechococcus*^57^ all of which were detected in samples during this pilot study. While cyanotoxin synthetase genes are an indicator of toxin-producing potential, they do not necessarily indicate the presence of a cyanotoxin^40^. This study could be repeated at multiple sites, adding additional toxins, and using identical sampling methods to help identify the cause of variations that may affect community composition, toxin gene abundance, or toxin concentrations.

### Relevance to Voyageurs National Park

Water-based activities occur throughout the day and night, but most water quality samples are collected during daylight hours. This incongruence makes collection of samples that truly represent exposure risk to people and pets a challenge. Our ultimate goal is to determine whether cyanotoxins are higher or lower during late evening and early morning hours (indicating greater or less risk to visitors). During this pilot, we focused on identification of the toxin producing species, toxin genes, and dissolved and total microcystin. Anglers in the region tend to fish during the early morning hours or at dusk, so this question is both environmentally and socially relevant and the pilot study is a first step toward guiding resource managers on whether or not extra precautions are necessary during certain times.

### Limitations

While this pilot study has its limitations, including coarse eDNA data, lack of repetition beyond the 24 h, limited toxin concentration data, and no replicate analysis between the grab and Van Dorn sampling methods, the results can guide us in our larger research effort. The difference between the two sites suggests against combining datasets from more than one location. These variances warrant a larger study to analyze conditions across a continuous bloom, during different times of the year, and for longer than 24 h.

## Conclusions

The novelty of this pilot study was that samples for cyanobacteria, cyanobacterial gene abundance, and microcystin were collected throughout a 24-hour period. We did not find any other research that sampled neurotoxin synthetase genes at night in a natural system. We sampled cyanotoxin gene abundance and cyanobacterial eDNA at 2 locations at a recurring bloom site in Kabetogama Lake, Voyageurs National Park, MN, USA. We sampled dissolved and total microcystin at one site. We measured photosynthetically active radiation, to assess the relation between sunlight, cyanobacteria, and microcystin.

We determined that sites must be considered individually for a 24-hour analysis. The phytoplankton and cyanobacteria at two nearby sites in the same bloom were significantly different in composition and biodiversity and this difference between sites (pseudo-F = 27.547, *p* = 0.001) was greater than the temporal difference over 24 h (pseudo F = 2.0938, *p* = 0.127) as revealed by nMDS and PERMANOVA analyses. Cyanotoxin synthetase genes had some day-to-night differences, but no significant increasing or decreasing trends and the day-to-night differences were smaller than the differences between sites. Additionally, the highest dissolved and total microcystin concentrations occurred at night. Further analysis of cyanotoxins may be necessary to understand the interplay between cyanobacterial species and their toxins. This pilot study is an important step to inform water resource managers on the differences between exposures on shoreline versus dock locations and the potential for cyanotoxin risk at night. As a pilot study, it also provides data informing a more comprehensive study of diel and spatial variation in cyanotoxins.

## Materials and methods

A recurring bloom site at the Ash River Boat Docks was selected (USGS site 482603092511801) for this pilot study based on historic presence of cyanobacterial blooms as well as with consideration for safety during low light navigation. Samples were collected during a 24-hour period on September 9 to 10, 2021, to capture the diel variation in phytoplankton community composition and toxin producing capabilities. Sampling occurred at two locations within the bloom: just below the surface at the lakeward end of a boat dock where water depth was approximately 2 m (site A) and at the surface along the adjacent shoreline (site B). The sites were located in a protected embayment and not subjected to wind or other disturbances. Wind speed on the day of sampling at the nearby International Falls Airport was 0 mph for most of the sampling period, with a few short episodes of up to ~ 5 miles per hour (generally from the SE).

Sampling consisted of (1) discrete field parameter measurement with a water-quality sonde and water transparency with a Secchi disk, (2) photosynthetically active radiation (PAR) with a hand-held PAR sensor, and (3) water collection for analysis of phytoplankton eDNA, toxin gene quantitative polymerase chain reaction (qPCR), and total and dissolved microcystins. The sampling process took place every three hours, beginning at 16:00 h and ending at 13:00 h the following day.

### Field measurements

Water chemistry measurements were recorded with a YSI EXO multi-probe sonde (Yellow Springs, Ohio). Field measurements included water temperature, specific conductance, pH, and dissolved oxygen concentration. Measurements were collected at a depth of 1 m at the dock (site A) and a depth of 0.1 m at the shoreline (site B). Secchi depth was recorded only at the dock (site A) due to insufficient water depth at the shoreline (site B).

### Photosynthetically active radiation

PAR is defined as the amount of radiation across a spectral range of 400 to 700 nm. Discrete measurements in this spectral range were made with a handheld PAR sensor (Vernier Software & Technology, Beaverton, OR). It was important to capture the sunlight reaching the water surface and not just the time of day, as some days are cloudier than others and time of day would not be a true representation of sunlight and thus potential photodegradation. The PAR sensor reports the Photosynthetic Photon Flux Density (PPFD), which is measured in micromoles of photons per meter squared per second. This measurement is the sum of light across the 400–700 nm range.

### Water sample collection

At the dock (site A), water samples were collected with a Van Dorn sampler which was triple rinsed in site water and then deployed to 1 m to capture sub-surface water. The samples were poured into a 1 L Nalgene composite bottle and then subsamples were transferred to 250 mL HDPE amber plastic bottles. At the shoreline (site B) a 1 L Nalgene composite bottle was filled directly from the surface using grab sampling procedures^26^. Subsamples were then transferred to 250 mL HDPE amber plastic bottles.

Field replicates were collected 3 times during the 24-hour period. For samples collected with the Van Dorn sampler, field replicates used the same water composite as the original sample (split replicates). For dip sampling replicates, two separate bottles were used (sequential replicates).

The bloom location was within a short walk of our lakeside laboratory (approximately 300 m), so all samples were processed or placed in a freezer or refrigerator within about 10 minutes of sampling.

### Sample Preparation and laboratory analysis

For environmental DNA analysis, frozen samples were thawed at room temperature, water was drawn into a 60 mL syringe, a 25 mm 1.0 μm pore-size Whatman nylon filter was attached, and the sample water was pushed through the filter and discarded. This process was repeated until the filter clogged, at which point 50 mL of air was pushed through the filter and Triton TE buffer preservative was injected into the filter. The filter was capped, sample information recorded, and shipped to Jonah Ventures (Boulder, CO) for environmental DNA (eDNA) analyses, bioinformatics, and quantitative polymerase chain reaction (qPCR) for cyanotoxin synthetase genes.

DNA extraction, sequencing, and bioinformatics followed Jonah Ventures workflows^58^. Briefly, at the commercial laboratory, genomic DNA was extracted from samples using DNeasy PowerLyzer PowerSoil Kits (Catalog number 12855-100) and the manufacturer’s protocol. For each sample, a portion of the chloroplast *trn*L intron was amplified via polymerase chain reaction (PCR) using a two-step protocol with the primers p23SrV_f1 and Diam23Sr1 (forward primer: GGACAGAAAGACCCTATGAA, reverse primer: TGAGTGACGGCCTTTCCACT)^59–61^. A second round of PCR was performed to complete the sequencing library construct, appending with the final Illumina (San Diego, CA) sequencing adapters and integrating a sample-specific,12-nucleotide index sequence. Indexed amplicons from each sample were cleaned and normalized using SequalPrep Normalization Plates (Life Technologies, Carlsbad, CA), purified and normalized using the Life Technologies SequalPrep Normalization kit (cat#A10510-01) according to the manufacturer’s protocol, and then pooled together. Sample library pools were sent for sequencing on an Illumina NovaSeq 6000 (San Diego, CA) at the Texas A&M Agrilife Genomics and Bioinformatics Sequencing Core facility using the SP Reagent Kit v1.5 (500 cycles) (cat# 20028402). Necessary quality control measures were performed at the sequencing center prior to sequencing.

Sequence data were demultiplexed using pheniqs v2.1.0^62^ and gene primers from the forward and reverse reads were removed using cutadapt v3.4^63^ before merging using vsearch v2.15.2^64^. For each sample, reads were clustered using the unoise3 denoising algorithm^65^ excluding sequences observed less than 8 times. Exact sequence variants (ESVs, amplicon sequences with 100% sequence identity) were compiled and potential chimeras removed using the uchime3 algorithm in vsearch. Taxonomy was assigned to ESVs using a custom best-hits algorithm and a reference database consisting of publicly available sequences from GenBank^66^ as well as Jonah Ventures voucher sequences records.

Samples analyzed for cyanotoxin synthetase genes followed Jonah Ventures workflows. Extracted DNA from each sample was analyzed by qPCR using assays for the cyanotoxins microcystin, anatoxin, and saxitoxin that each used unique probe and primer sets. All qPCR quantification assays were performed on a QuantStudio 5 qPCR instrument (Applied Biosystems, Waltham, MA, USA). The *mcyE* assay used a SYBR green probe and microcystin primers (Forward primer: 5’-TTTGGGGTTAACTTTTTTGGGCATAGTC-3’, reverse primer: 5’-AATTCTTGAGGCTGTAAATCGGGTTT-3’^67^). The *anaC* assay used a SYBR green probe and anatoxin primers (Forward primer: 5’ TCTGGTATTCAGTCCCCTCTAT 3’, reverse primer: 5’ CCCAATAGCCTGTCATCAA 3’^68^). The *sxtA* assay used a saxitoxin-specific fluorescein-based probe and saxitoxin primers (Forward primer: 5’ GGAGTGGATTTCAACACCAGAA 3’, reverse primer: 5’ GTTTCCCAGACTCGTTTCAGG 3’, probe: 5’ /56-FAM/TGCCGATTTAGAAGAAAGTATCCTCTCAG/3IABkFQ/ 3’^69^). Microcystin and anatoxin assays used Absolute QPCR Mix, SYBR Green, Low ROX (Thermo Scientific, Waltham, MA, USA) and saxitoxin assays used PerfeCTa qPCR ToughMix, Low ROX (Quantabio, Beverly, MA, USA). Each cyanotoxin gene assay used an associated calibration curve based on a series of seven triplicated, 10-fold dilutions of cyanotoxin standards of known concentration. Linear regression was used to assess the relation between the log10-transformed standard concentration and the number of PCR cycles at which the detection threshold was reached (Cq).

For toxins, whole water samples were extracted from the Nalgene bottle, placed into 125 mL HDPE bottles, and frozen within minutes of collection. For the filtered samples, 10 mL was extracted from the Nalgene bottle, filtered through 0.7-µm syringe filters^70^ and placed into 125 mL brown HDPE bottles. Samples were analyzed for dissolved and total microcystins by enzyme-linked immunosorbent assay (ELISA) at the USGS Organic Geochemistry Research Laboratory (Lawrence, KS, USA). Cells were lysed by subjecting water samples to three sequential freeze-thaw cycles^70^. Gold Standard Diagnostic ELISA kits were used to measure microcystin (detection limit 0.1 µg/L). The USGS Organic Geochemistry Research Laboratory performed multiple quality-control checks on these measurements including the laboratory replicates on each sample, blind spiked samples, and an assessment of inter-assay variability.

### Quality control and quality assurance

To establish data reliability, we conducted specific procedures to ensure that samples accurately represented the water body being studied. To accompany our environmental data, we processed three replicate samples: two from the end of the boat dock and one at the shoreline. The relative percent difference (RPD) was calculated for each eDNA sequence detected in the sample, then averaged to get an average RPD for each sample. All three replicate samples were similar to their respective environmental samples since the average RPD was less than (< ) 15% for each sample.

When splitting field replicates for cyanobacterial composition, gene abundance, and toxin concentration, high variation is expected because it is difficult to homogenize intact cells. For the samples analyzed for toxin concentration, additional field replicate samples were not reanalyzed when % relative standard deviation (RSD) exceeded 28.3%. Rather, this variability was accepted as inherent due to both temporal and spatial variation caused by sample splitting^71^. All quality- control data for this pilot study were considered acceptable.

### Data compilation and statistical analysis

Taxonomy was initially assigned to organisms based on agreement between the sample sequence and reference sequence percent matches. Consensus taxonomic resolution was removed for each rank in 5% increments of sequence percent match (≥ 95% match assigned to species, 90–94.9% match assigned to genus). To simplify unresolved taxonomy and aid in interpretation, we further resolved the taxonomy of organisms following the approach of Schulte et al.^58 ^for some visualizations and analyses. Briefly, we assigned the reference sequence taxonomy associated with the highest percent match to each ESV in our dataset, regardless of the initial consensus taxonomy assigned to the organisms. In cases where the ESV assigned to an organism corresponded to multiple reference sequences with the same highest percent match, we assigned the most frequent genus out of the tied reference sequences. There were thirteen instances where the assigned ESV and highest percent match of reference sequences corresponded to Class-level taxonomy (all for Bacillariophyceae). We removed all non-algal or non-cyanobacterial ESVs from our dataset before any figure generation or analyses.

We used Friedman tests^72^ as a nonparametric alternative to ANOVA to assess differences in cyanotoxin synthetase gene abundances over time. We analyzed cyanotoxin gene abundances at each location (dock or shoreline) separately, in part because of the orders-of-magnitude differences in gene abundances between locations. When statistically significant differences (*p* < 0.05) among sampling times were detected, we used Dunn tests to determine the sampling times that differed from one another. Given the many pairwise comparisons among sampling times, Benjamini-Hochberg corrections were used in an effort to control false discovery rate. Friedman and Dunn tests were conducted in R (v.4.4.0)^73^ using the function ‘friedman.test()’ in base R, and the function ‘dunnTest()’ in package ‘FSA’^74^.

Primer-e software^41^ was used to calculate nMDS, construct shade plots, and perform PERMANOVA analyses. Briefly, data was transformed with square root to reduce the importance of the very abundant taxa and to take into account less-common taxa. Bray-Curtis similarity index^75^ was then calculated, using 100 restarts and a minimum stress of 0.01 (analyzed on samples in Fig. 6). The PERMANOVA analysis within Primer-e was used to present the pseudo-F statistic and p-value to determine whether significant differences existed between location or time^75^.

## Data Availability

The datasets generated and/or analyzed during the current study are available in the USGS Sciencebase^76^ data repository, at [https://doi.org/10.5066/P13Q4EJ9]. Additionally, all field measurements and toxin concentrations are available from the USGS at [https://waterdata.usgs.gov/download-samples/#monitoringLocationIdentifier=USGS-482603092511801&startDate=2021-09-09&endDate=2021-09-10&dataProfile=fullphyschem].
